# 

**Published:** 1864-12

**Authors:** 


					﻿Volume XXI.
Number
THE CHICAGO
MEDICAL JOURNAL
De 1 ;kie miller, m. d.,
ELT JM 1NGALS, M. D.,
itopa and Proprietors.
DECEMBER, 1^64.
CHICAGO :
J. 0. W. BA1L1T, BOOK AND JOB PR1NTBK, IN CLARK JTRMT.
1864.
				

